# Ageing of Immune System and Response to a Live-Attenuated Herpes Zoster Vaccine in Lung Transplant Candidates

**DOI:** 10.3390/vaccines9030202

**Published:** 2021-02-28

**Authors:** Lei Wang, Erik A.M. Verschuuren, Davy Paap, Christien Rondaan, Elisabeth Raveling-Eelsing, Siqi Liu, Johanna Westra, Nicolaas A. Bos

**Affiliations:** 1 University Medical Center Groningen, Department of Rheumatology and Clinical Immunology, University of Groningen, 9713 EZ Groningen, The Netherlands; l.wang@umcg.nl (L.W.); d.paap@umcg.nl (D.P.); e.raveling-eelsing@umcg.nl (E.R.-E.); s.liu@umcg.nl (S.L.); johanna.westra@umcg.nl (J.W.); 2 University Medical Center Groningen, Department of Pulmonary Diseases and Tuberculosis, University of Groningen, 9713 EZ Groningen, The Netherlands; e.a.m.verschuuren@umcg.nl; 3 University Medical Center Groningen, Department of Rehabilitation Medicine, University of Groningen, 9713 EZ Groningen, The Netherlands; 4 University Medical Center Groningen, Department of Medical Microbiology and Infection Prevention, University of Groningen, 9713 EZ Groningen, The Netherlands; c.rondaan@umcg.nl

**Keywords:** lung transplantation, ageing, herpes zoster vaccine, age associated B cells

## Abstract

The mean age of lung transplant recipients has significantly increased in recent decades. Elderly recipients have a higher risk of developing herpes zoster (HZ), and they have in general a worse response to vaccination than younger persons do. We investigated the relationship between the humoral and cellular immune response to a live-attenuated HZ vaccine (Zostavax^®^, Merck Sharp and Dohme) and the frequencies of T and B cell subsets, especially aged cell subsets (CD28−T cells and age associated B cells, ABCs). In total, 37 patients awaiting lung transplantation received one dose of Zostavax^®^, and peripheral blood was collected before and within 6 months after vaccination. We observed a robust immune response after vaccination. The frequencies of CD28−T cells before vaccination had no impact on the subsequent immune response to HZ vaccination. However, a higher frequency of ABCs before vaccination correlated with a lower immune response especially regarding the cellular immune response. Cytomegalovirus seropositivity was associated with increased frequencies of CD28−T cells but not with frequencies of ABCs in the patients. In conclusion, increased levels of ABCs might disturb the cellular immune response to HZ vaccination, which could lower the efficacy of such vaccination in elderly transplant recipients.

## 1. Introduction

Lung transplantation is a final treatment for end-stage pulmonary disease (ESPD), providing an improved quality of life and survival benefit for the patients. The number of lung transplants has greatly increased in recent decades because of advances in the selection processes for recipients and donors, improvement of the surgical techniques, and knowledge of immunosuppression [1]. Every year, around 4000 lung transplantations are performed worldwide [2]. With the ageing of the population, the age of lung transplant recipients is also increasing. From 2006 to mid-2012, 10% of the lung transplant recipients were older than 65 years, approximately 3 times higher than in the years from 2000 to 2005 [3]. Despite the fact that age has no longer been considered an absolute restriction for transplantation, elderly recipients do have a lower survival rate after lung transplantation than younger recipients [4]. The median survival was 3.6 years in patients older than 65 years, while it was 6.5 years in patients aged 35–49 years [3]. Age was a significant risk factor for mortality, and graft failure, infection, and malignancy contribute significantly to the mortality. Interestingly, elderly recipients had a lower incidence of acute rejection, but they did have higher infection rate than younger recipients, and both could be caused by the declined immune system function with ageing, termed immunosnescence [5].

Studies have shown that cellular subset distribution shifts from naïve cells to memory cells during ageing, accompanied by decreased expression of co-stimulatory surface receptors such as CD28 [6]. In addition, a mature B cell subset pool, named age associated B cells (ABCs), was also found to be significantly enlarged with increasing age [7]. These shifts lead to lower proliferation of immune cells, eventually causing re-occurrence of previously controlled infections, such as with varicella zoster virus (VZV) [6]. VZV is a human α-herpes virus that establishes lifelong latency in dorsal root ganglia after primary infection (chickenpox). Reactivation of VZV causes herpes zoster (HZ), which is often complicated with postherpetic neuralgia, a pain lasting more than 3 months after the onset of HZ [8]. Lung transplant patients are faced with a high risk of developing HZ due to their age and continuously high-dose immunosuppression. A live-attenuated HZ vaccine (Zostavax^®^, Merck Sharp and Dohme, Kenilworth, NJ, USA) was shown to reduce the incidence of HZ by 61.1% in a randomized, double-blind study that enrolled 38,546 adults over 60 years old [9]. However, the efficacy significantly decreased with age and was only 18% in adults over 80 years old [10]. In addition, the vaccine was only recommended to be given at least 4 weeks before transplantation due to the risk for live vaccine-induced infections [11].

Impairment of the immune system caused by immunosenescence not only enhances the risk for infection but also restricts the efficacy of vaccination [6]. It has been reported that response to influenza vaccination, including production of neutralizing antibodies, antibody repertoire diversity, and CD4+ and CD8+ cellular responses, is reduced in older people [12]. Although several studies investigating the VZV-specific immune responses in elderly people have been performed, evidence for how immunosenescence affects the response to HZ vaccine is still lacking [12,13]. In our study, patients with ESPD awaiting lung transplantation were given HZ vaccination, and frequencies of their T- and B cell subsets, especially aged cell subsets (CD28−T cells and ABCs), were investigated. We aimed to assess if there were any correlations between immune response to the HZ vaccine and the ageing status of the immune system before vaccination.

## 2. Materials and Methods

### 2.1. Study Design and Participants

At the University Medical Center Groningen (UMCG), all VZV seropositive ESPD patients who were newly screened for lung transplantation were given 1 dose of Zostavax^®^ (Merck Sharp and Dohme, Kenilworth, USA). The efficacy and safety of this vaccination strategy has been reported previously [14]. Blood was drawn from vaccinated adult patients who were willing to participate in this study before vaccination and within 6 months (range 0.7–5.8 with median 2.2 months) after vaccination. Baseline characteristics of the included patients are shown in Table 1. Blood was taken, and peripheral blood mononuclear cells (PBMCs) were isolated immediately after collection. In brief, fresh blood was diluted with RPMI 1640 [LONZA (Basel, Switzerland), supplemented with 1% gentamicin] and isolated by density gradient centrifugation using lymphoprep (Alere Technologies Inc, Oslo, Norway). PBMCs were stored in liquid nitrogen, and serum was stored at −20 °C until use. Upon thawing, cell viability was evaluated by trypan blue staining and was between 85% and 100%. 

### 2.2. Assessment of Humoral Immune Response to HZ Vaccination

The humoral immune response to HZ vaccination was quantitatively evaluated by an in-house glycoprotein (gp) VZV enzyme-linked immunosorbent assay (ELISA) as previously described [15]. VZV purified glycoproteins (EastCoastBio, North Berwick, ME, USA) were used as antigen, and pooled human serum with known levels of anti-gpVZV was used as standard. VZV-IgG levels higher than 100 mIU/mL were defined as positive according to the recommendations of Institute Virion/Serion.

### 2.3. Assessment of Cellular Immune Response to HZ Vaccination

The VZV-specific cell-mediated immune response (CMI) to HZ vaccination was evaluated by an interferon (IFN)-γ enzyme-linked immunospot (ELISpot) assay as previously described [15]. Briefly, a MultiScreen filter plate (Merck Millipore, Burlington, VT, USA) was coated overnight at 4 °C with 50 µL of anti-human IFN-γ (Mabtech, Nacka Strand, Sweden). Then, 2 × 10^5^/per well PBMC suspension was added and stimulated with 10 µL 1:14 pre-diluted UV-inactivated Zostavax^®^ (>19,400 plaque-forming units/0.65 mL), 5 µg/mL concanavalin A (positive control) or only culture medium (negative control). All assessments were performed in duplicate except for the positive control. After 48 h of incubation, the plates were stained and dried, and spots were counted using an AID ELISpot Reader (Autoimmun Diagnostika GmbH, Straßberg, Germany). The mean number of the spots of negative control wells was subtracted from the mean number of corresponding VZV-stimulated wells. The number of spots represented the number of IFN-γ spot-forming cells (SFCs) per 2 × 10^5^ PBMCs.

### 2.4. Flow Cytometry

Flow cytometry was performed using an LSR II flow cytometer (Becton Dickinson, Franklin Lakes, NJ, USA). To analyze T cell subsets, PBMCs were thawed and stained (1.0 × 10^6^ cells/100 µL) with antibodies specific for CD3 (Biolegend, San Diego, CA, USA; 317344), CD4 (BD biosciences, San Jose, CA, USA; 345769), CD8 (BD biosciences, 345772), CD25 (Biolegend, 356128), CD127 (BD biosciences, 742547), CXCR5 (BD biosciences, 564624), CD45RA (BD biosciences, 562886), CCR7 (BD biosciences, 557648), PD1 (BD biosciences, 565299), and CD28 (Biolegend, 302948) for 60 min. T cell subsets (CD3+) were defined as follows within CD4+ or CD8+ compartments: naïve cells (CCR7+/CD45RA+), central memory cells (CM, CCR7+/CD45RA−), effector memory cells (EM, CCR7−/CD45RA-), terminally differentiated cells (TD, CCR7−/CD45RA+), follicular T helper cells (Tfh, CD25−CD127low/−/CXCR5+/PD1mod/+), regulatory T cell (Treg, CD4+/CD25+/CD127low/−), and senescent T cells (CD28−). During the whole procedure light exposure was avoided.

As for analysis of B cell subsets, PBMCs were thawed and stained (1.0 × 10^6^ cells/100 µL) with antibodies specific for CD19 (BD biosciences, 563325), CD27 (Biolegend, 356418), CD38 (Biolegend, 356616), IgD (BD biosciences, 348228), IgM (Biolegend, 314532), CD11c (Biolegend, 337206), and CD21 (BD biosciences, 564437) for 60 min. B cell subsets were defined as follows within the CD19+ compartment: naïve cells (CD27−/CD38−), transitional cells (CD27−/CD38+), plasma blast/plasma cells (PB/PC, CD27+/CD38+), memory cells (CD27+/CD38−), and age associated B cells (ABCs, CD21−/CD11c+). Within the memory cells, subsets were further defined as follows: switched memory cells, (IgM−/IgD−), non-switched memory cells (IgM+/IgD+), IgM only memory cells (IgM+/IgD−). During the whole procedure light exposure was avoided.

### 2.5. Statistical Analyses

Flow cytometry data were processed with the software Kaluza (Beckman Coulter, Brea, CA, USA). To compare data within a group before and after vaccination, the Wilcoxon signed-rank test was used. Data of different subgroups were compared using the Mann–Whitney test. Spearman’s rho was used for correlations. The frequencies of T and B cell subsets were normalized to z-scores using the means and standard deviation of the total values before and after vaccination and are presented in the heat maps. A two-tailed *p*-value less than 0.05 was regarded as statistically significant. All data were analyzed using Prism 8 for Windows (GraphPad Software, San Diego, CA, USA) and SPSS Statistics (version 23, IBM, Armonk, NY, USA).

## 3. Results

### 3.1. Patient Characteristics and Safety of HZ Vaccine

The characteristics of the participants included in this study are shown in Table 1. In total, 37 patients received one dose of Zostavax^®^ between November 2016 and February 2019. The primary ESPD was chronic obstructive pulmonary disease/emphysema (51.4%). Most of the patients were older than 45 years old, and three patients were at a young age at time of vaccination (19, 26, and 33 years old). Sixteen of these patients underwent lung transplantation, with a median of 13.2 months after HZ vaccination. In addition, the number of cytomegalovirus (CMV) seropositive and seronegative patients was very similar (19 and 18, respectively).

One patient experienced swelling and erythema at the injection site after vaccination. No other side effects or episodes of HZ were reported during the study period. A total of eight patients died while waiting for or after lung transplantation. None of these deaths were related to HZ vaccination.

### 3.2. Aged T and B Cell Subsets before Vaccination

To assess the baseline immune status of patients, we performed flow cytometry on PBMCs collected at baseline. We analyzed the median frequencies of naïve (52.7%), EM (21.3%), CM (20.6%), and TD (3.8%) T cells within CD4+T cells and of naïve (25.4%), EM (28.2%), CM (2.8%), and TD (38.3%) T cells within CD8+T cells. The baseline B cell subsets were characterized as naïve (70.6%), transitional (2.7%), PB/PC (1.3%), or memory (22.7%) B cells within the CD19+ cells. Furthermore, the subsets of memory B cells were determined as isotype-switched (18.4%), non-switched (75.5%), or IgM only (0.5%) memory B cells (Table 2 and Table A1). The changes in those subsets after vaccination are discussed below in Section 3.7.

**Figure 1:**
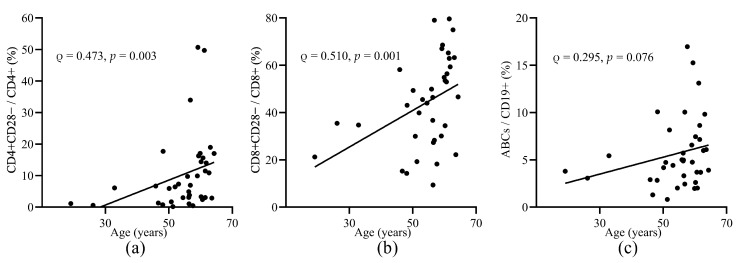
The correlation of the frequencies of aged T and B cell subsets and age before vaccination. Correlation of the frequencies of CD4+CD28−T cells (**a**), CD8+CD28−T cells, (**b**) and ABCs (**c**) and age of the patients before vaccination. ABCs, age associated B cells. Spearman’s rho and *p*-values are shown.

To determine the immunosenescence status, we focused on the frequencies of aged T cells (CD28−) and aged B cells (ABCs) before vaccination (Table 2 and Table A1). We investigated whether there was a correlation between the frequencies of the aged cell subsets and the age of the patients before vaccination. The frequencies of CD4+CD28− and CD8+CD28− cells were both correlated with age before vaccination (ρ = 0.473, *p* = 0.003 and ρ=0.510, *p* = 0.001, respectively) (Figure 1a,b). There was no significant correlation between ABCs frequency and age of the patients in our study (Figure 1c).

### 3.3. Humoral and Cellular Immune Response to HZ Vaccine

**Figure 2:**
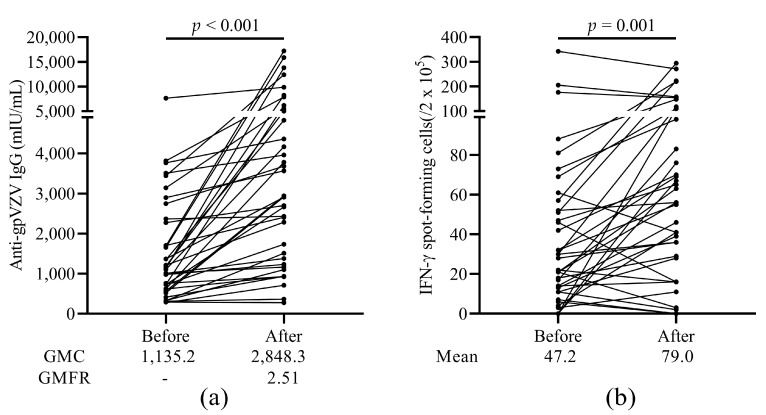
Humoral and cellular immunogenicity of the HZ vaccine. (**a**) Levels of anti-glycoprotein (gp) VZV IgG antibody. GMC and GMFR are shown under the figure. (**b**) The number of VZV stimulated IFN-γ spot-forming cells (SFCs) before and after vaccination. HZ, herpes zoster; gp, glycoprotein; VZV, varicella zoster virus; GMC, geometric mean concentrations; GMFR, geometric mean fold rise.

To evaluate the humoral and cellular immune response to HZ vaccination, we performed an in-house gp-VZV ELISA and ELISpot assay, respectively. As shown in Figure 2, we detected an increased immune response in most of the patients after vaccination. VZV-IgG geometric mean concentration (GMC) was 2,848.3 mIU/mL, which was a 2.51 (95% confidence interval, CI: 1.97–3.20; *p* < 0.001) geometric mean fold rise (GMFR) (Figure 2a). As for the cellular immune response to vaccination, the number of VZV-specific IFN-γ SFCs was significantly increased after vaccination (*p* = 0.001, Figure 2b). We calculated the delta changes of VZV-IgG levels or VZV SFC numbers before and after vaccination of each patient. One patient had a decreased VZV-IgG level (289.0 to 276.7 mIU/mL). Ten patients had reduced numbers of VZV SFCs after vaccination compared to before vaccination. Of interest, we found a significant positive correlation between VZV-IgG levels and VZV SFC numbers before and after vaccination (ρ = 0.425, *p* < 0.001).

### 3.4. The Relationship between Response to HZ Vaccination and Baseline T and B Cell Subsets

To study whether cellular frequencies of subsets in patients before vaccination affected the immune response to HZ vaccination, we divided the patients based on their delta change of VZV-IgG levels below or above the median level. The same was done for the delta change of VZV SFC numbers. The frequencies of all T and B cell subsets at baseline in these groups are shown in Table A2. Patients with high delta change of VZV-IgG levels had significantly higher percentages of CD8+ cells (*p* = 0.022), lower percentages of CD4+ (*p* = 0.011) and EM CD8+ cells (*p* = 0.024), as well as lower CD4+/CD8+ ratios (*p* = 0.019) at baseline. Patients with high delta change of VZV SFC numbers showed significantly higher percentages of transitional B cells (*p* = 0.030) at baseline.

### 3.5. The Relationship between Response to HZ Vaccination and Baseline Ageing Cell Subsets

In order to investigate the possible influence of aged immune cells at baseline on the vaccination response, we divided the patients into subgroups according to frequencies of ageing subsets (CD28−T cells or ABCs) lower or higher than the median level at baseline.

**Figure 3:**
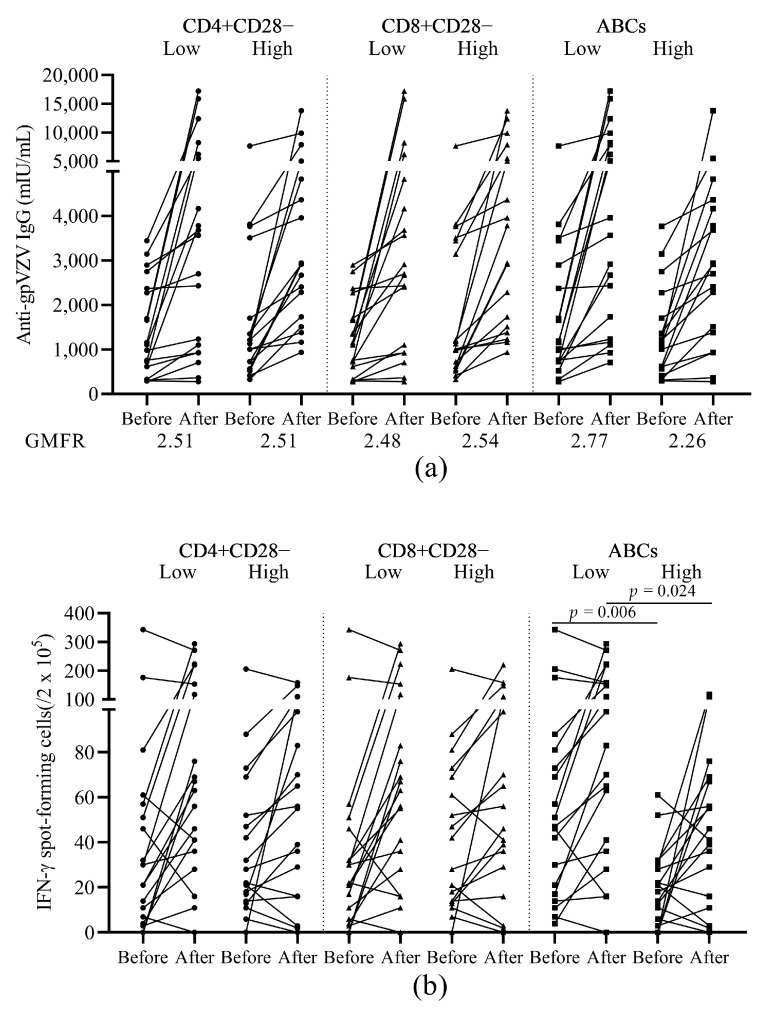
Humoral and cellular immunogenicity of the HZ vaccine in patients with low or high baseline frequencies of ageing cell subsets. (**a**) Levels of anti-glycoprotein (gp) VZV IgG antibody before and after vaccination. GMFR after vaccination over before vaccination is shown under the figure. (**b**) The number of VZV stimulated IFN-γ secreting T cell spots before and after vaccination. HZ, herpes zoster; gp, glycoprotein; VZV, varicella zoster virus; GMFR, geometric mean fold rise; ABCs, age associated B cells. Patients were divided into subgroups according to their baseline frequencies of ageing subsets (CD4+CD28−, CD8+CD28−, or ABCs) lower or higher than the median level.

Patients with higher percentage of ABCs at baseline had relatively lower GMFRs (2.77 vs. 2.26, respectively) in VZV-IgG levels (Figure 3a). These patients with high baseline ABCs percentages also showed lower VZV SFC numbers both before (*p* = 0.006) as well as after vaccination (*p* = 0.024) compared to the patients with lower percentage of ABCs at baseline (Figure 3b). We did not observe any changes in response to vaccination for either the humoral or the cellular immune response when we compared patients with high and low percentages of aged T cell subsets (CD28−) (Figure 3a,b).

### 3.6. The Relationship of HZ Vaccination Response and CMV Latency

**Figure 4:**
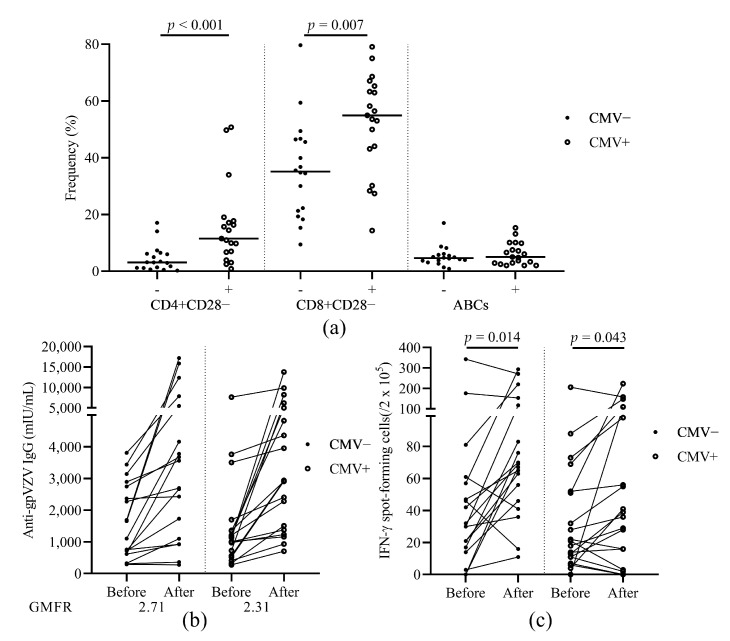
The effect of CMV serostatus on aged T/B cell subsets and immunogenicity of the HZ vaccine. (**a**) The frequencies of CD4+CD28−, CD8+CD28−, and ABCs before vaccination. (**b**) Levels of anti-glycoprotein (gp) VZV IgG antibody before and after vaccination. GMFR after vaccination over before vaccination is shown under the figure. (**c**) The number of VZV stimulated IFN-γ secreting T cell spots before and after vaccination. HZ, herpes zoster; gp, glycoprotein; VZV, varicella zoster virus; GMFR, geometric mean fold rise; ABCs, age associated B cells; cytomegalovirus, CMV. Patients were divided into subgroups according to their CMV serostatus.

To study the role of CMV latency in immunosenescence and vaccination response, we compared the frequencies of aged T and B cells before vaccination and also the humoral and cellular immune response in patients according to CMV serostatus (Figure 4a). CMV seropositive patients showed significantly higher frequencies of aged CD4+CD28− and CD8+CD28− cells before vaccination (Figure 4a). We also compared the humoral and cellular immune response to HZ vaccine within patients according to CMV serostatus. Patients with CMV seropositivity had higher VZV-IgG GMFR (2.71 vs. 2.31, Figure 4b) but similar VZV-SFCs (Figure 4c) compared to CMV seronegative patients.

### 3.7. Changes in T and B Cell Subsets after Vaccination

**Figure 5:**
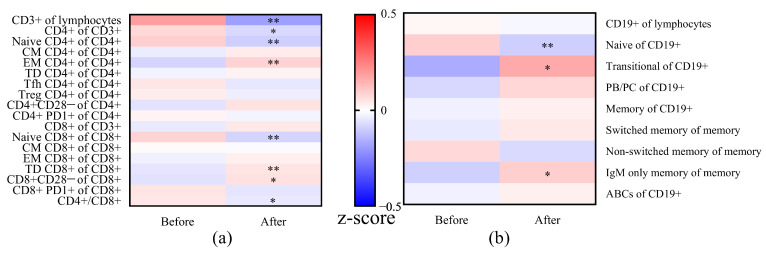
Comparison of frequencies of T (**a**) and B (**b**) cell subsets in patients before and after vaccination. The frequencies were normalized to z-scores, using the means and standard deviation per subset (values before and after vaccination as the total group). The normalized z-scores were displayed on a color scale, ranging from −0.5 (blue means before vaccination or after vaccination lower than the means of the total) to 0.5 (red means before vaccination or after vaccination higher than the means of the total). The frequencies of T/B cell subsets were compared between before vaccination and after vaccination using the Wilcoxon signed-rank test. * *p* < 0.05 and ** *p* < 0.01. Abbreviations: CM, central memory; EM, effector memory; TD, terminally differentiated; Tfh, T follicular helper cells; Treg, regulatory T cell; PB/PC, plasma blast/plasma cell; ABCs, age associated B cells.

Next, we compared the changes in the distribution of T cell subsets in patients before and after vaccination (Figure 5 and Table 2). A significantly decreased percentage of CD4+ cells (*p* = 0.029) was observed after vaccination, while the percentage of CD8+ cells increased, which was also reflected in the significantly decreased CD4+/CD8+ ratio (*p* = 0.046). Significantly decreased frequencies of naïve cells were seen in both CD4+ and CD8+ cells while observing significantly elevated frequencies of EM in CD4+ and TD in CD8+ cells after vaccination. As for the aged T cells, higher frequencies of CD28− cells were found especially in CD8+ cells (*p* = 0.012) after vaccination compared to the frequencies before vaccination.

As for the B cell subsets, a significantly lower frequency of naïve B cells and higher frequency of transitional and IgM only memory B cells was seen within 1 to 6 months after vaccination. We did not find a difference in frequency in ABCs before and after vaccination.

## 4. Discussion

In this study, patients with ESPD awaiting lung transplantation received one dose of a live-attenuated HZ vaccine. We observed robust immune responses to this vaccine in most of the patients. The baseline frequencies of CD28−T cells had no relation to the subsequent humoral and cellular response to HZ vaccination. There was, however, a correlation between the frequency of ABCs at baseline and the cellular response to vaccination. CMV seropositivity was associated with the loss of expression of CD28 on T cells, but no relation to frequencies of ABCs and CMV serostatus in the patients was seen.

A decreased VZV-specific cellular response to vaccination was seen in 27% of patients in our study, although overall significantly higher numbers of VZV-SFCs were observed after vaccination. Weinberg et al. reported peak values of VZV IFN-γ SFCs one week after vaccination following by a rapid decline later [16], and a similar tendency was also seen in other studies [17,18,19]. Our blood samples were collected with a median of 2.2 months after vaccination, and the optimal time point to detect the peak cellular response was possibly missed. Contrary to cellular responses, only one patient showed a decrease in VZV-IgG titer after vaccination. This patient did have a low baseline VZV-IgG level, and studies have suggested that pre-vaccination VZV immunity affects immunogenicity to HZ vaccine [19,20], which might explain the reduced VZV-IgG level in this patient. Although abundant evidence has shown that cellular immunity is key to preventing HZ, humoral immune response has been seen to correlate with HZ protection [21]. The statistical criterion for an acceptable VZV-IgG GMFR is that the lower bound of the two-sided 95% CI should be at least 1.4 according to previous studies [22]. In our study, the GMFR value after vaccination showed a median of 2.51, with 1.97 in the lower bound of the two-sided 95% CI, meeting the acceptability criterion and being consistent with the results of HZ vaccination in a healthy population [18].

Since several studies have shown that pre-vaccination immunity affected the quantitative response to HZ vaccine in the elderly [16,20], we assessed the frequencies of T and B cell subsets before vaccination, with a focus on the aged subsets, to investigate the relationship between pre-existing immunosenescence and HZ vaccine response. CD28 is an important co-stimulatory marker of effector CD4+ and CD8+ T cells [23], and reduced expression of CD28 was found to be related to immunosenescence, accompanied by lessened replicative lifespan and reduced proliferative ability in antigenic challenge. These functional disturbances may contribute to the compromised immune response to vaccination in the elderly, as shown for influenza vaccination [24]. However, in our cohort, we failed to observe correlations between the loss of CD28 and both humoral and cellular immune response to HZ vaccine, although the frequencies of CD4+CD28− and CD8+CD28− did significantly increase with age of the patients. It is possible that our ESPD patient group was not aged enough to show the effect of increasing CD8−T cells on the vaccination response, since the oldest patient was 64 years of age.

B cells are crucially important in terms of the humoral immune response. ABCs, with expression of CD11c, CD11b, and T-bet and lack of CD21 and CD23 as primary criterion, were first defined by Hao et al. and Rubstov et al. independently in 2011 [25,26]. ABCs were found to accumulate in elderly people, and they showed “late memory B cell” features including impaired ability to produce antibodies and low telomerase activity. Here, we found that patients with a high baseline frequency of ABCs exhibited a lower VZV-IgG GMFR after vaccination, which was consistent with a study on the influenza vaccine that reported diminished antibody response related to the expansion of ABCs population [27]. Interestingly, we also found significantly lower number of VZV-SFCs before and after vaccination in patients with a high frequency of ABCs. ABCs were found to be enriched in some autoimmune and autoinflammatory diseases and might contribute to an increase in overall inflammatory cytokines [7]. It is possible that the inflammatory microenvironment caused by increased ABCs may have disturbed the T cell response to HZ vaccine in our study.

CMV is a β-herpes virus that commonly infects 60–90% of older adults. CMV infection is usually asymptomatic but can cause severe diseases in immunocompromised patients. The persistent subclinical challenge of latent CMV leads to senescence and exhaustion of the immune system [28]. It is also reported that CMV latency is associated with a decreased immune response to influenza vaccination [29,30]. In our study, CMV seropositivity was related to an increase of CD28−T cells but not of ABCs in the ESPD patients. As described above, more ABCs correlated with a lower immune response to the HZ vaccination, while aged T cells did not. The role of CMV positivity in HZ vaccination in our study was not conclusive.

The frequencies of T and B cell subsets before and after vaccination were compared. Although there was a shift from naive to memory cells after vaccination, which could be features of immunosenescence [6], we could not draw a firm conclusion that this was caused by HZ vaccination due to the lack of control groups. We could only analyze total T and B cell subsets and not VZV-specific T and B cell subsets because of the low frequency of VZV-specific subsets. The frequencies of T and B cell subsets are only relative, and absolute counts were not determined in our study. These are the limitations of our study. Patients received medications to control their respiratory problems or other issues after vaccination. The influence of medication on vaccination response and profile of T and B cell subsets could not be investigated due to the complexity of the medical status of each patient. Furthermore, a multilevel analysis was performed to investigate if frequencies of cell subsets or other parameters could predict the immune response to the vaccine. Due to the limited number of patients and the relatively small variance of age, no conclusive results were found. Although HZ episodes were observed later on in our study, the effect of vaccination on the incidence of HZ episodes could not be evaluated due to the short follow up time. Currently, there are several strategies for improving efficacy of HZ vaccination in the elderly. One is administration of a booster dose of the HZ vaccine. Levin et al. reported significantly enhanced VZV-specific cellular response in people older than 70 years with a second dose of HZ vaccine 10 years after the first dose [18]. Another way to improve the immunogenicity of the HZ vaccine is through introducing adjuvants. In 2017, a recombinant subunit vaccine (Shingrix^®^, GlaxoSmithKline, Brentford, UK) containing VZV gE with the AS01_B_ adjuvant system was approved by the Food and Drug Administration, and it showed 97% efficacy in preventing HZ in adults older than 50 years. The adjuvant system probably offered a remarkable protection for HZ in older individuals, although it also raises moderate reactogenicity [10,31]. Currently, there is no study of the use of Shingrix^®^ in ESPD patients, but a systematic literature review showed acceptable safety and immunogenicity of this vaccine in immunocompromised patients [32].

## 5. Conclusions

Overall, we observed robust immune responses to Zostavax^®^ in most ESPD patients awaiting lung transplantation, and our data suggested that increased levels of ABCs might disturb the cellular immune response to HZ vaccination. This is still an exploratory study with a relatively small size, but our observations do support and extend the current knowledge about the influence of immunosenescence on HZ vaccination in vulnerable patient groups. Further studies are needed regarding the increase of ABCs in elderly patient groups on HZ vaccine response and how this can also affect T cell immunity.

## Figures and Tables

**Table 1:** Characteristics of the participants at baseline.

Characteristics	N = 37
Age at Vaccination in Years, Median (Range)	56.8 (19.1–64.3)
Months After Vaccination, Median (Range)	2.2 (0.7–5.8)
Gender, N (%)	
Male	20 (54.1)
Female	17 (45.9)
End Stage Pulmonary Disease, N (%)	
Chronic Obstructive Pulmonary Disease/Emphysema	19 (51.4)
α_1_-Antitrypsin Deficiency	9 (24.3)
Pulmonary Fibrosis/Interstitial Lung Disease	3 (8.1)
Pulmonary Arterial Hypertension	2 (5.4)
Cystic Fibrosis/Bronchiectasis	4 (10.8)
Baseline Cytomegalovirus Serostatus, N (%)	
+	19 (51.4)
–	18 (48.6)
Age at Vaccination, N (%)	
≤50 Years Old	7 (18.9)
50–60 Years Old	18 (48.6)
>60 Years Old	12 (32.4)
Follow Up Time After Vaccination in Months, Median (Range)	23.7 (9.3–36.8)
Lung Transplantation During Follow Up, N (%)	16 (43.2) ^1^
Months After Vaccination at Transplantation, Median (Range)	13.2 (2.8–29.6)

^1^ All patients underwent bilateral lung transplantation except one vaccinated patient who underwent liver–lung transplantation.

**Table 2:** Frequency of T and B cell subsets in the lung transplant candidates before and after vaccination.

Subsets	Before	After	*p*-Value
CD3+/Lymphocytes (%)	73.8 (66.8–81.2)	70.7 (62.1–76.9)	<0.001
CD4+/CD3+ (%)	67.4 (58.3–75.8)	65.8 (54.0–73.2)	0.029
Naïve CD4+/CD4+ (%)	52.7 (28.7–61.3)	45.6 (31.3–57.7)	<0.001
EM CD4+/CD4+ (%)	21.3 (17.2–30.7)	24.3 (20.4–33.6)	<0.001
Naïve CD8+/CD8+ (%)	25.4 (11.8–40.4)	20.4 (9.5–36.2)	0.002
TD CD8+/CD8+ (%)	38.3 (21.4–59.0)	38.8 (23.8–60.0)	0.005
CD8+CD28−/CD8+ (%)	45.5 (29.2–58.8)	52.9 (25.0–62.1)	0.012
CD4+/CD8+ Ratio	2.7 (1.7–4.2)	2.3 (1.5–3.6)	0.046
Naive CD19+/CD19+ (%)	70.6 (59.7–76.2)	67.4 (60.2–70.0)	0.007
Transitional CD19+/CD19+ (%)	2.7 (1.4–6.2)	4.9 (1.8–7.4)	0.032
IgM Only Memory CD19+/Memory CD19+ (%)	0.5 (0.3–0.9)	0.6 (0.4–1.0)	0.029

Abbreviations: SFCs, spot-forming cells; CM, central memory; EM, effector memory; TD, terminally differentiated; Tfh, T follicular helper cells; Treg, regulatory T cell; PB/PC, plasma blast/plasma cell; ABCs, age associated B cells. Median (interquartile range) of the percentage is shown. Only significant data are presented, and full data are provided in Table A1 in Appendix A.

## Data Availability

The data presented in this study are available on request from the corresponding author. The data are not publicly available due to privacy reasons.

## Appendix A

**Table A1:** Frequency of T and B cell subsets in the lung transplant candidates before and after vaccination.

Subsets	Before	After	*p*-Value
CD3+/Lymphocytes (%)	73.8 (66.8–81.2)	70.7 (62.1–76.9)	<0.001
CD4+/CD3+ (%)	67.4 (58.3–75.8)	65.8 (54.0–73.2)	0.029
Naïve CD4+/CD4+ (%)	52.7 (28.7–61.3)	45.6 (31.3–57.7)	<0.001
CM CD4+/CD4+ (%)	20.6 (13.1–28.3)	20.4 (15.3–28.3)	ns
EM CD4+/CD4+ (%)	21.3 (17.2–30.7)	24.3 (20.4–33.6)	<0.001
TD CD4+/CD4+ (%)	3.8 (2.7–7.8)	4.6 (2.0–8.0)	ns
Tfh CD4+/CD4+ (%)	15.9 (11.4–22.1)	16.2 (10.7–21.2)	ns
Treg CD4+/CD4+ (%)	6.5 (5.2–7.8)	6.5 (5.1–8.3)	ns
CD4+CD28−/CD4+ (%)	6.5 (2.7–15.0)	7.5 (2.5–16.5)	ns
CD4+PD1+/CD4+ (%)	1.5 (1.0–2.1)	1.8 (1.1–2.2)	ns
CD8+/CD3+ (%)	24.7 (17.8–34.2)	28.9 (20.4–35.9)	ns
Naïve CD8+/CD8+ (%)	25.4 (11.8–40.4)	20.4 (9.5–36.2)	0.002
CM CD8+/CD8+ (%)	2.8 (1.5–5.8)	2.8 (1.6–5.4)	ns
EM CD8+/CD8+ (%)	28.2 (15.4–37.2)	27.3 (17.3–40.3)	ns
TD CD8+/CD8+ (%)	38.3 (21.4–59.0)	38.8 (23.8–60.0)	0.005
CD8+CD28−/CD8+ (%)	45.5 (29.2–58.8)	52.9 (25.0–62.1)	0.012
CD8+PD1+/CD8+ (%)	1.0 (0.6–2.0)	0.9 (0.6–1.6)	ns
CD4+/CD8+ Ratio	2.7 (1.7–4.2)	2.3 (1.5–3.6)	0.046
CD19+/Lymphocytes (%)	3.8 (2.3–5.5)	3.8 (2.4–4.9)	ns
Naive CD19+/CD19+ (%)	70.6 (59.7–76.2)	67.4 (60.2–70.0)	0.007
Transitional CD19+/CD19+ (%)	2.7 (1.4–6.2)	4.9 (1.8–7.4)	0.032
PB/PC CD19+/CD19+ (%)	1.3 (1.0–2.3)	1.7 (1.1–2.8)	ns
Memory CD19+/CD19+ (%)	22.7 (15.7–32.4)	24.4 (17.3–33.2)	ns
Switched Memory CD19+/Memory CD19+ (%)	18.4 (11.2–24.1)	17.8 (12.4–25.0)	ns
Non-Switched Memory CD19+/Memory CD19+ (%)	75.5 (65.2–81.1)	73.2 (66.8–81.4)	ns
IgM Only Memory CD19+/Memory CD19+ (%)	0.5 (0.3–0.9)	0.6 (0.4–1.0)	0.029
ABCs/CD19+ (%)	4.9 (3.0–7.3)	4.4 (2.9–7.8)	ns

Abbreviations: CM, central memory; EM, effector memory; TD, terminally differentiated; Tfh, T follicular helper cells; Treg, regulatory T cell; PB/PC, plasma blast/plasma cell; ABCs, age associated B cells. Median (interquartile range) of the percentage is shown. *p*-value more than 0.05 was regarded as non-statistically significant (ns).

**Table A2:** Frequency of T/B cell subsets before vaccination in patients with low/high delta changes of VZV-IgG or VZV-SFCs.

Frequencies before Vaccination	VZV-IgG ^a^			VZV-SFCs ^a^		
	Low (N = 19)	High (N = 18)	*p*-Value	Low (N = 19)	High (N = 18)	*p*-Value
CD3+/Lymphocytes (%)	75.6 (66.5–80.5)	73.3 (67.3–82.6)	ns	76.8 (66.7–82.0)	72.5 (66.8–76.7)	ns
CD4+/CD3+ (%)	71.9 (64.8–78.1)	61.7 (54.0–67.5)	0.011	67.9 (53.1–78.1)	66.9 (60.0–74.2)	ns
Naïve CD4+/CD4+ (%)	56.8 (24.2–63.3)	47.4 (34.7–56.1)	ns	47.4 (27.0–61.6)	55.4 (34.5–61.2)	ns
CM CD4+/CD4+ (%)	17.8 (12.7–26.2)	22.8 (13.8–28.8)	ns	22.2 (12.7–28.9)	18.8 (13.0–26.8)	ns
EM CD4+/CD4+ (%)	18.7 (14.1–37.0)	23.0 (19.8–29.8)	ns	21.3 (17.9–37.0)	19.7 (14.0–25.6)	ns
TD CD4+/CD4+ (%)	4.2 (2.7–6.6)	3.5 (2.4–9.3)	ns	4.2 (2.8–8.9)	3.3 (1.6–7.6)	ns
Tfh CD4+/CD4+ (%)	15.3 (10.9–21.7)	16.8 (12.1–23.0)	ns	17.4 (10.9–21.9)	14.8 (11.7–22.4)	ns
Treg CD4+/CD4+ (%)	5.9 (4.9–7.7)	6.8 (5.5–8.0)	ns	6.3 (4.8–7.8)	6.6 (5.5–8.0)	ns
CD4+CD28−/CD4+ (%)	3.9 (1.7–16.3)	6.8 (3.1–12.5)	ns	9.9 (2.4–17.0)	6.1 (2.6–10.8)	ns
CD4+PD1+/CD4+ (%)	1.4 (0.9–1.9)	1.6 (1.2–2.8)	ns	1.3 (0.9–2.0)	1.6 (1.2–2.3)	ns
CD8+/CD3+ (%)	20.6 (16.3–27.9)	30.6 (23.5–35.2)	0.022	21.4 (16.4–35.8)	25.7 (21.0–33.2)	ns
Naïve CD8+/CD8+ (%)	22.5 (6.6–31.6)	27.2 (14.1–46.5)	ns	19.3 (9.0–41.7)	27.8 (15.4–415)	ns
CM CD8+/CD8+ (%)	3.9 (1.8–6.4)	2.6 (0.9–4.5)	ns	2.8 (1.7–5.9)	2.5 (0.9–5.0)	ns
EM CD8+/CD8+ (%)	34.0 (24.3–47.0)	23.1 (13.2–30.5)	0.024	28.3 (13.4–38.5)	27.2 (18.8–37.7)	ns
TD CD8+/CD8+ (%)	38.3 (21.6–50.6)	38.7 (20.7–59.4)	ns	38.5 (24.1–61.6)	36.3 (19.5–58.9)	ns
CD8+CD28−/CD8+ (%)	36.8 (22.2–59.4)	46.6 (34.1–59.4)	ns	46.7 (28.3–65.2)	44.8 (28.1–56.9)	ns
CD8+PD1+/CD8+ (%)	1.0 (0.6–2.4)	1.0 (0.5–1.7)	ns	0.8 (0.5–1.5)	1.1 (0.7–2.4)	ns
CD4+/CD8+ Ratio	3.7 (2.3–4.7)	2.0 (1.6–2.9)	0.019	3.3 (1.5–4.5)	2.6 (1.9–3.6)	ns
CD19+/Lymphocytes (%)	3.7 (2.2–5.8)	4.1 (2.3–5.6)	ns	4.5 (2.0–5.8)	3.8 (2.3–5.6)	ns
Naive CD19+/CD19+ (%)	66.5 (59.9–81.2)	72.6 (58.4–75.5)	ns	70.6 (55.4–81.2)	71.1 (59.8–75.5)	ns
Transitional CD19+/CD19+ (%)	2.1 (1.6–5.2)	3.6 (1.2–7.0)	ns	1.9 (0.6–3.6)	4.4 (2.1–7.0)	0.030
PB/PC CD19+/CD19+ (%)	1.2 (0.7–2.8)	1.6 (1.3–2.2)	ns	1.2 (0.9–2.8)	1.4 (1.2–2.2)	ns
Memory CD19+/CD19+ (%)	25.1 (15.8–32.8)	21.6 (15.4–31.8)	ns	25.0 (15.8–36.8)	21.6 (15.4–30.2)	ns
Switched Memory CD19+/Memory CD19+ (%)	20.3 (11.9–25.2)	17.6 (9.5–20.6)	ns	19.5 (11.9–32.0)	16.7 (9.5–20.7)	ns
Non-Switched Memory CD19+/Memory CD19+ (%)	75.5 (63.8–81.0)	76.0 (69.8–81.6)	ns	72.4 (62.4–80.6)	77.4 (68.3–82.5)	ns
IgM Only Memory CD19+/Memory CD19+ (%)	0.4 (0.3–0.6)	0.8 (0.4–1.3)	ns	0.5 (0.3–1.3)	0.5 (0.3–0.9)	ns
ABCs/CD19+ (%)	4.9 (3.3–7.5)	4.3 (2.9–7.4)	ns	5.0 (3.1–8.6)	4.6 (2.9-5.8)	ns

Abbreviations: SFCs, spot-forming cells; CM, central memory; EM, effector memory; TD, terminally differentiated; Tfh, T follicular helper cells; Treg, regulatory T cell; PB/PC, plasma blast/plasma cell; ABCs, age associated B cells. ^a^ Patients were divided into two groups according to the changes (values after vaccination subtracted by values before vaccination) of VZV-IgG levels or VZV-SFC numbers lower or higher than the median level. Median (interquartile range) of the percentage is shown. *p*-value more than 0.05 was regarded as non-statistically significant (ns).
